# Antibacterial potential of a new glucosaminidase from *Pediococcus acidilactici* ITV26 against Gram-negative and Gram-positive pathogens

**DOI:** 10.3389/fmicb.2025.1728482

**Published:** 2025-12-29

**Authors:** Karla G. Alvarez-Villagomez, Daniel Balleza, Carolina Peña-Montes, Itzel C. Fonseca-Barrera, Patricia G. Mendoza-García

**Affiliations:** Unidad de Investigación y Desarrollo en Alimentos, TecNM/Instituto Tecnológico de Veracruz, Veracruz, Mexico

**Keywords:** peptidoglycan (PG) hydrolases, *N*-acetylglucosaminidase (*N*AGase), autolysis, enzybiotics, *Pediococcus acidilactici*

## Abstract

**Introduction:**

*Pediococcus acidilactici* ITV26 is a homofermentative lactic acid bacterium capable of producing pediocins that restrict the proliferation of pathogenic bacteria. However, these bacteria have also been described to produce several peptidoglycan hydrolases (PGH) with great antibacterial potential. These enzymes have been classified as enzybiotics, i.e., proteins capable of degrading cell walls in a highly specific manner to killing pathogens.

**Methods:**

Through differential extraction of protein fractions associated with the membrane of this bacterial strain and in silico analysis, we report the compositional, physicochemical, and biochemical characterization of an enzyme with N-acetylglucosaminidase (NAGase) activity. Additionally, we determined the bactericidal activity of this enzymatic extract against two Gram-positive and two Gram-negative pathogens. We also evaluated ultrastructural changes in sensitive bacteria using scanning electron microscopy (SEM).

**Results:**

This NAGase activity shows a high specificity against Micrococcus lysodeikticus ATCC 4698 cell walls. The active protein has a molecular mass close to 110 kDa, a sequence of 935 residues, a possible transmembrane fragment close to the N-terminal end, and a profile of high intrinsic flexibility with high hydrophilicity, consistent with a possible topology for a peripheral membrane protein. The enzyme induces significant autolytic activity in *P. acidilactici* under acidic conditions and is strongly active against Staphylococcus aureus ATCC 6538 and ATCC 25923, *Clostridium perfrigens* ATCC 12916, Klebsiella pneumoniae ATCC 10031 and *Pseudomonas aeruginosa* ATCC 25619.

**Discussion:**

This NAGase has great potential as an enzybiotic, as an alternative to the use of conventional antibiotics. Showing little hemolytic activity, this protein has pharmacological potential against multidrug-resistant bacteria. We propose a mechanism of action in which this enzyme hydrolyzes GlcNAc units present in the cell wall of diverse pathogens.

## 1 Introduction

The alarming rise of antimicrobial resistance (AMR) represents one of the most pressing global health challenges of our time, threatening the efficacy of conventional antibiotic treatments. Microbial pathogens capable of “escaping” the action of beta-lactamic and no-lactamic antibiotics, particularly ESKAPE microbes (*Enterococcus faecium*, *Staphylococcus aureus*, *Klebsiella pneumoniae*, *Acinetobacter baumannii*, *Pseudomonas aeruginosa*, and *Enterobacter* species), underscore the urgent need for innovative and effective antimicrobial strategies to deal with this emerging pandemic. The World Health Organization (WHO) estimates that around 700,000 people worldwide die each year due to AMR, and also predicts that these pathogens could cause up to 10 million deaths per year worldwide by 2050 (Mancuso et al., 2021; World Health Organization, 2022; Algammal et al., 2023). In such circumstances, the search for new antibiotics is a constant priority. Given that the most common mechanisms of resistance to conventional antibiotics (i.e., penicillins, *β*-lactams, cephalosporins, carbapenems, aminoglycosides, tetracyclines, chloramphenicol, fluoroquinolones, and sulfonamides, among others) include the expression of specific enzymes capable of degrading such compounds, enzybiotics have recently been chosen as a very promising class of novel antibiotics.

Since their discovery in bacteriophages, enzybiotics have been defined as proteins with endolysin activity, capable of degrading the bacterial cell wall as part of their lytic cycle. These endolysins are highly active hydrolases, as they exhibit high specificity in hydrolyzing the peptidoglycan (PG) layer of pathogenic microorganisms without killing beneficial microbiota, in addition to having a low probability of developing AMR (Dams and Briers, 2019; Rahman et al., 2021). However, this bactericidal property is not exclusive to viral endolysins, as another group of proteins of bacterial origin (autolysins) has been described as an analogous group of enzymes involved in the controlled remodeling of the cell wall during cell growth and division. Thus, these bacterial hydrolases exhibit potent antimicrobial (enzybiotic) activity by depolymerizing PG chains when applied exogenously (Vermassen et al., 2019). Phage therapies have recently been implemented as alternatives to the use of antibiotics (Lin et al., 2017), while the use of bacterial autolysins is comparatively less widespread. Given their endogenous origin, high specificity, and low risk of generating AMR, these bacterial enzybiotics are a promising therapeutic tool in scientific research and development of new antimicrobial agents.

In lactic acid bacteria (LAB) reports are rather scarce, although different research groups have described high molecular weight PG hydrolases (PGHs). The group of LAB plays a key role in human nutrition because it is assumed that these bacteria have coevolved with humans since the beginning of early agricultural practices (Shibata and Yagin, 1996). Furthermore, as part of the evolutionary strategies of LAB, their enzyme system encodes bacteriocins and PGHs, which enable them to compete against potentially harmful microbiomes present in the human digestive system. Thus, the search for and characterization of these enzyme systems is very promising as a source of novel antibiotics in the context of human health.

*Pediococcus acidilactici,* are Gram-positive bacteria generally recognized as safe (GRAS) and widely used in food fermentation and as probiotics. These bacteria naturally possess sophisticated cell wall degradation systems, primarily mediated by endogenous PGHs, a process known as autolysis (García-Cano et al., 2011; Todorov et al., 2023). The inherent ability of these enzymes to degrade bacterial cell walls makes LAB an excellent reservoir for the discovery of novel PGHs with antimicrobial potential (Mora et al., 2003; Najjari et al., 2016). In this context, the aim of this work is to elucidate the nature of a PGH with a putative glucosaminidase activity from *P. acidilactici* ITV26, highlighting its molecular weight and lytic capacity, as well as exploring its potential as a novel antimicrobial agent against a wide range of bacterial pathogens.

## 2 Materials and methods

### 2.1 Peptidoglycan hydrolase (PGH) producing strain and growth conditions

The molecular and biochemical identification of *P. acidilactici* ITV26 has been reported elsewhere (López Zamudio et al., 2021). In this work, bacterial cultures and growth kinetics were carried out in MRS broth (glucose 20 g L^−1^, sodium acetate 5 g L^−1^, K_2_HPO 2 g L^−1^, ammonium citrate 2 g L^−1^; MgSO_4_ 0.2 g L ^1^, MnSO_4_ 0.04 g L^−1^, casein peptone 10 g L^−1^, meat extract 8 g L^−1^, extract yeast 4 g L^−1^ and Tween 80 1 mL L^−1^) at 37 ± 2 °C. The harvest for the inoculation of the kinetic assay was performed when bacterial cultures reached an Optical Density of 0.2–0.4 (OD_600_).

### 2.2 Cell autolysis assay in buffer solution

Autolysis activity was assessed by assays with whole cells of *P. acidilactici* ITV26 grown to the exponential phase (OD_600nm_ 1–1.5). Bacterial cells were collected by centrifugation at 3,000 × g for 10 min at 4 °C and washed twice with 50 mmol L^−1^ potassium phosphate buffer, pH 6.5, 50 mmol L^−1^ potassium phosphate buffer, pH 6.5 with Triton-100 ×. For the control, cells were heat-denatured in a water bath at 100 °C for 15 min, centrifuged 10 min at 3,000 × g at 4 °C, and resuspended in mmol L^−1^ potassium phosphate buffer, pH 6.5. This initial optical density reading was recorded at time 0 (T0). Subsequently, the suspension was incubated at 30 °C, with measurements taken every 12, 24, 48, and 96 h. The percentage of autolysis was determined following the method outlined by Nájera-Domínguez and Gutiérrez-Méndez (2013), using the formula:


$$\text{Autolysis}\;(\%)=(\frac{{\mathrm{OD}}_{600\mathrm{nm}}\;\text{Inicial}-{\mathrm{OD}}_{600\mathrm{nm}}\;\mathrm{at}\;\text{time}\;t}{{\mathrm{OD}}_{600\mathrm{nm}}\text{inicial}})\times 100$$

### 2.3 Extraction of the membrane-bound proteins

An aliquot (1 mL) was taken from the bacterial culture of *P. acidilactici* ITV26, which was grown in MRS broth for 18 h. Once an optical density (OD_**600**_) of 1.5 ± 0.05 was reached, the biomass was collected by centrifugation at 8000 × g for 10 min, 4 °C. The resulting cell pellet was washed twice with distilled water and resuspended in SDS-extraction solution [10 mM Tris HCl pH 8.0, 10 mM ethylenediaminetetraacetic acid (EDTA), 10 mM NaCl, 2% (w/v) SDS]. The suspension was vortexed for 5 s with glass beads, boiled for 5 min, and then centrifuged at 10,000 × g for 20 min at 4 °C. SDS was removed from the soluble fraction following the methodology. Finally, the sample was concentrated using Amicon Ultra-15 centrifugal filter units (UFC901926; Merck-Millipore) with a 50 kDa size exclusion limit membrane by centrifugation at 4,000 × g for 30 min. The protein concentration in the final sample was determined by the assay of Bradford (1976) using the Quick start Bradford Protein Assay (Bio-Rad).

### 2.4 *β*-*N*-acetylglucosaminidase (*N*AGase) activity assay

The *N*AGase enzyme activity was monitored using 4-nitrophenyl *N*-acetyl-D-glucosaminide (4-NP-Glc*N*Ac, Sigma**−**Aldrich) as a substrate analog to *N*-acetylglucosamine (Glc*N*Ac) according Shibata and Yagin (1996). The reaction was carried out in a citric acid monohydrate buffer (50 mM at pH 5). To quantify the enzyme product, *p*-nitrophenol (*p*-NP), a standard curve was prepared using 0–60 μmol of 4-NP-Glc*N*Ac. The standards were treated with a 100 mM NaCO_3_ solution to stop the reaction. The hydrolyzed *p*-NP was quantified by measuring the absorbance at 410 nm. The enzymatic reaction was prepared in quintuplicate in 96-well microtiter plates, containing 10 μL of 4-NP-Glc*N*Ac and 40 μL of the protein sample recovered at different time points every 3 h from the start of cell growth, during a period of 21 h. Blanks (substrate and enzyme) and a positive control (*β*-*N*-acetylglucosaminidase from the plant *Canavalia ensiformis*) (Sigma**−**Aldrich) were included. After 30 min of incubation at 37 °C, the reaction was stopped by adding 100 μL of NaCO_3_ (140 mM). The net absorbance of the reaction (∆A_410_) was calculated by subtracting the average absorbance of the blanks (substrate and enzyme) from the reaction samples. The units of enzymatic activity (U/mL) were calculated using the following formula:


$$\text{Activity}\;(\mathrm{Um}L^{-1})=\frac{\Delta A410\times F\;}{\left(\text{Tmin})\times (\mathrm{VmL}\right)}$$

where $\mathrm{Um}L^{-1}$ is the volumetric activity; F: the reciprocal of the slope of the standard curve that converts the observed absorbance into μmol of product; Tmin: reaction time, in minutes; VmL: volume of the enzyme sample used in the reaction, in mL.

### 2.5 Antimicrobial activity of PGH ITV26 and hemolytic activity

The spectrum of activity of the PGH from *P. acidilactici* ITV26 was tested against several clinically important pathogens obtained from the American Type Culture Collection (ATCC; St. Cloud, MN). This included four Gram-positive bacteria: *Staphylococcus aureus* (ATCC 6538), *S. aureus* (ATCC 25923), *Clostridium perfringens* (ATCC 12916), and *Listeria monocytogenes* (ATCC 35152); and five Gram-negative bacteria: *Escherichia coli* (ATCC 8739), *K. pneumoniae* (ATCC 10031), *Salmonella typhimurium* (ATCC 14028), *P. aeruginosa* (ATCC 25619), and *P. aeruginosa* (ATCC 25923) by the agar well diffusion method (Tagg et al., 1976). The antibiotic sensitivity pattern of these pathogens was evaluated using the multidisc PT-34 (Multibac I.D., Mexico), which contains the following antibiotics: ampicillin (10 μg), penicillin (10 μg), dicloxacillin (1 μg), pefloxacin (5 μg), tetracycline (30 μg), ceftazidime (30 μg), gentamicin (10 μg), erythromycin (15 μg), cefotaxime (30 μg), trimethoprim/sulfamethoxazole (25 μg), cefuroxime (30 μg), and cephalothin (30 μg). Antimicrobial activity was determined by the presence of an inhibition zone around the growth of the evaluated pathogens. To determine the hemolytic activity of this PGH, the *P. acidilactici* ITV26 strain was tested on blood agar plates containing 5% (v/v) lamb blood and incubated at 37 °C for 24–48 h. The results were registered as follows: *α* hemolysis, presence of a green coloration around the colonies; *β*-hemolysis, clear and transparent around the colonies; and *γ*-hemolysis, no color.

### 2.6 SDS-PAGE and zymography

Sample preparation was performed as described by Lortal et al. (1997). A concentration of 48.14 ± 0.89 μg/mL of each sample was resuspended in 2 × loading buffer [3.55 mL deionized water, 1.25 mL 0.5 M Tris–HCl (pH 6.8), 2.5 mL glycerol, 2.0 mL 10% (w/v) SDS, and 0.5% (w/v) bromophenol blue]. SDS-PAGE electrophoresis was carried out using a 10% polyacrylamide separating gel in a Mini-Protean Tetra Cell, 4-Gel system. For size estimation, 10 μL of Precision Plus Protein Dual Xtra Standars (M.W. 2–250 kDa) was used. Running voltages were set to 80 V for the stacking gel and 120 V for the separating gel. After electrophoresis, SDS-PAGE bands were observed following the method of Llorente-Bousquets et al. (2008). Zymogram gels, containing 0.2% (w/v) autoclaved *Micrococcus lysodeikticus* ATCC 4698 cells as the substrate, were prepared and analyzed as determined by García-Cano et al. (2011) and Shibata and Yagin (1996).

### 2.7 Mass spectrometry-based protein identification

Prior to LC–MS/MS analysis, the Coomassie blue-stained SDS-PAGE gel band exhibiting activity on zymograms was excised into ~1 mm^3^ cubes for chemical-enzymatic digestion. The in-gel digestion protocol was performed as described by Rivas-Mercado et al. (2025) using dithiothreitol (DTT) for reduction, iodoacetamide (IAA) for alkylation, and trypsin solution for enzymatic digestion. The resulting peptides were desalted using ZipTip C18 (Millipore) and concentrated in a Savant SPD1010 SpeedVac (Thermo Fisher Scientific). Finally, the peptides were resuspended in 20 μL of 0.1% formic acid in Milli-Q water to obtain the final peptide mixture.

### 2.8 LC–MS/MS analysis

Peptides were analyzed using an LC–MS/MS system (Vanquish Neo UHPLC system coupled to an Orbitrap Ascend mass spectrometer, Thermo Fisher Scientific). Peptides were separated on an Acclaim PepMap C18 column (Thermo Fisher Scientific) using a 24-min gradient with solvent A (0.1% formic acid in water) and solvent B (0.1% formic acid in acetonitrile), starting from 0% solvent B and increasing to 99% solvent B. A constant flow rate of 0.350 μL/min was maintained. All spectra were acquired in positive ion mode. The mass spectrometry acquisition method was configured with an m/z range of 375–1,500. Full MS scans were performed at an Orbitrap resolution of 120,000 FWHM at m/z 200, with a dynamic exclusion duration of 30 s. For fragmentation, Higher-Energy Collisional Dissociation (HCD) was employed with a normalized collision energy (NCE) of 30%.

### 2.9 *In silico* analysis of PGH protein sequence

Raw MS data were processed using Proteome Discoverer version 1.4 and searched against UniProt databases for *Pediococcus* spp. and *Lactiplantibacillus plantarum*. Search parameters included a fragment mass tolerance of 0.60 Da, a parent mass tolerance of 20 ppm, carbamidomethylation of cysteine as a fixed modification, and deamidation (asparagine/glutamine) and oxidation (methionine) as variable modifications. The protein sequence analysis was performed using ProtParam and ProtScale (Gasteiger et al., 2005), GlycoPP 1.0 (Chauhan et al., 2012); NetPhosBac 1.0 (Miller et al., 2009); ScanSite 4.0 (Obenauer et al., 2003); DAS (Cserzö et al., 1997), and FlexiProt (García-Morales et al., 2024) bioinformatic tools. The MAFFT online server was used for alignment and phylogenetic tree construction (Katoh et al., 2019).

### 2.10 Scanning electron microscopy (SEM)

Morphological changes in the cells of four of the bacteria studied (*K. pneumoniae* ATCC 10031, *S. aureus* ATCC 6538, *P. aeruginosa* ATCC 25923, and *C. perfringens* ATCC 12916) after treatment with or without the PGH-rich extract ITV26 were analyzed by SEM following the method of Varela-Pérez et al. (2022) with some modifications regarding the voltage used and the drying treatment. Bacterial cultures in the exponential growth phase (10^6^ CFU/mL) were harvested by centrifugation (6,000 rpm, 10 to 4 °C) and washed twice with PBS saline solution. Untreated cells (without PGH) were fixed by streaking directly onto carbon tape using a bacteriological loop. Cells in presence of 48 ± 1 μg/mL PGH were incubated at 37 ± 2 °C for 2 h. Following incubation, the sample was fixed onto carbon tape. The fixed cells were subsequently dried overnight in a glass desiccator with a hygroscopic substrate. Each sample was observed under a MIRA3 scanning electron microscope (Tescan®, Brno, Czech Republic) at an acceleration voltage of 8 kV under low vacuum, using a secondary electron detector at a working distance of 9.2 mm.

## 3 Results and discussion

### 3.1 Protein identification

*Pediococcus acidilactici* ITV26 was originally reported as isolated from the human neonatal microbiota (López del Castillo, 1998) and it has been characterized as probiotic (Santos et al., 2011). Considering a previous characterization of two lytic enzymes of *Pediococcus acidilactici* ATCC 8042 with molecular masses of 110 and 99 kDa (García-Cano et al., 2011), we explore the presence of these proteins in the *P. acidilactici* ITV26, where we have already reported the presence of a class IIa bacteriocin (Pediocin) which exhibits lytic activity (Calderón-Santoyo et al., 2001).

In this work, we identified an ortholog PGH protein produced by *P. acidilactici* ITV26 isolated from fermented foods and human neonate feces. The bacterial strain was initially identified with 96.3% identity using the API 50 CHL biochemical system. This initial phenotypic identification was complemented and confirmed genotypically via 16S ribosomal sequencing, showing high molecular identity (>98%) with *P. acidilactici* (López Zamudio et al., 2021). Furthermore, the phenotypic profile was extended by determining the antibiogram profile and studying the growth kinetics, with special attention on monitoring the phase of highest lytic activity. *P. acidilactici* ITV26 is susceptible to Tetracycline (TE), Penicillin (PE), Erythromycin (E), Clindamycin (CLM), Cefotaxime (CFX), Cephalothin (CF) and Ampicillin (AM), weakly resistant Gentamicin (GE) and resistant vancomycin (VA), Trimetoprim (SXT), Ciprofloxacin (CPF) and Dicloxacillin (DC), whereas the PGH activity described here was detected in the stationary phase, starting at 14 h of bacterial growth (Supplementary Figure S1). Given this information, we then determined that at 18 h of growth, the enzymatic activity was optimal for characterizing its antibacterial effects.

**Figure 1:**
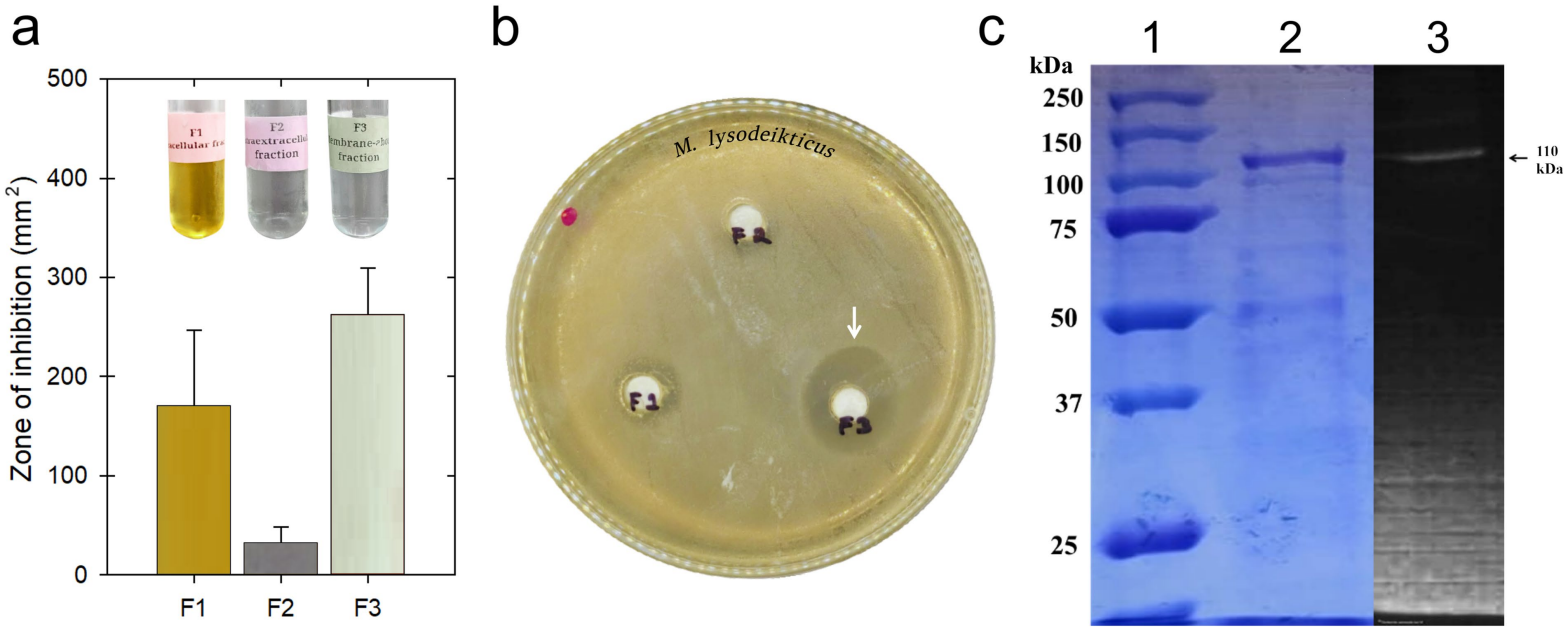
**(a)** Evaluation of the antibacterial potential of PGH-enriched extracts obtained from the extracellular (F1), intracellular (F2), and membrane-enriched (F3) fractions against *Micrococcus lysodeikticus* ATCC 4698. **(b)** Bacterial growth inhibition halos of the three PGH extracts from *Pediococcus acidilactici* ITV26 in a Petri dish with LB agar inoculated with *M. lysodeikticus* ATCC 4698. The membrane-rich F3 fraction is the most active. **(c)** Native PAGE and zymogram analysis of the F3 PGH extract from *P. acidilactici* ITV26 using *M. lysodeikticus* ATCC 4698 cell walls as substrate. 1: Molecular weight marker; 2: electrophoretic profile obtained in membrane-enriched (F3) fraction stained with Coomassie Brilliant Blue R-250; 3: zymography for PGH activity.

Then, aiming to obtain an enriched membrane-associated PGH and fully characterizing its enzymatic activity, we tested three methods of extracting the membrane-enriched fraction. First, we tested a method that we refer here as C1 (Chapot-Chartier and Kulakauskas, 2014), then we tested the G1 method (Gandhi et al., 2020). After evaluating both results, we decided to use a modified C1 method which includes the use of glass beads and vortexing with 3 cycles of 30 s each. Under these conditions, we obtained a concentration of 48.14 μg/mL, compared to 41.03 μg/mL and 13.84 μg/mL when using the original methods, C1 and G1, respectively. Thus, we determined that the extract enriched in the membrane fraction (F3) of *P. acidilactici* ITV26 was the most active in terms of the lytic activity against *M. lysodeikticus* ATCC 4698 (Figure 1). *M. lysodeikticus* is typically used as the standard substrate due to its extreme sensitivity to this enzyme (Salton, 1958). This high susceptibility is a direct consequence of its cell wall structure, since, as a Gram-positive bacterium, its thick layer of PG is directly exposed to the medium. More specifically, this kind of PG is characterized by a composition that lacks chemical modifications found in other bacteria (Salton, 1952).

Once demonstrated the lytic activity in the membrane-enriched fraction (F3), we determined the protein profile and the molecular weight of the proteins in F3 by sodium dodecyl sulphate-polyacrylamide gel electrophoresis (SDS-PAGE) and through the cell wall cleavage assay (zymogram) by using *M. lysodeikticus* ATCC 4698 as the substrate (Figure 1c). These results demonstrate that in the fraction enriched in the membrane proteins of *P. acidilactici* ITV26, a protein close to 110 kDa is the most abundant in this extract, which is also active against *M. lysodeikticus*. Thus, the convergence of results obtained through SDS-PAGE and the corresponding zymogram provides a strong basis for the identification of a PGH with a putative glucosaminidase activity in *P. acidilactici* ITV26 as the one previously reported by García-Cano et al. (2011).

Pediococcal PGHs are described in some previous reports, and they have molecular weights of approximately 99 and 110 kDa, being described as proteins with two catalytic domains, i.e., *N*-acetylmuramidase and *N*-acetylglucosaminidase activities (Mora et al., 2003; García-Cano et al., 2011). Given that PG is composed of alternating units of Glc*N*Ac and *N*-acetylmuramic acid (Mur*N*Ac) joined by *β*1 → 4 linkages, an enzyme with glucosaminidase activity is able to specifically hydrolyze *N*-acetyl-β-glucosamine residues in that substrate. Therefore, the combined evidence from molecular weight characterization and the specific lytic activity against *M. lysodeikticus*, in accordance with previous reports of the PGH from *P. acidilactici* with marked *N*-acetylglucosaminidase activity, firmly supports the hypothesis that the characterized enzyme in *P. acidilactici* ITV26 is an ortholog enzyme with glucosaminidase activity.

Previously, we characterized a pediocin in this bacterial strain, which has a molecular mass of 4.62 kDa and a pI close to 9 (Fraga-Chávez et al., 2007). We have also obtained enzyme extracts in three different lactic acid bacteria (LAB), one particularly effective against *S. aureus* ATCC 6538 and which is present in the membrane-associated fraction of *P. acidilactici* SP-50 (Supplementary Figure S2). Given this enzymatic diversity present in the LAB we analyzed, in this study we decided to characterize in detail the PGH present in the ITV26 strain by also determining its amino acid sequence. Thus, we determined the sequence of the ~110 kDa protein shown in Figure 1c, which exhibited strong hydrolase activity against *M. lysodeikticus*. Supplementary Figure S3 shows the sequence of this protein present in strain ITV 26, as well as four homologous proteins present in other strains and deposited in protein sequence databanks (Genbank, UniProt). The protein from strain ITV 26 includes 935 residues and shows 99.5% identity in 927 overlapping residues with the protein sequence present in strain ATCC 8042 reported by Shibata and Yagin (1996). As expected, this protein has two catalytic domains, i.e., *N*-acetylmuramidase and *N*-acetylglucosaminidase, separated by an Asn-rich region of about 400 residues, which is highly disordered (*data not shown*). Likewise, this PGH ITV26 has a theoretical pI of 9.23 and a theoretical molecular weight of 100.39 kDa. This molecular weight is slightly lower than expected based on the electrophoresis results shown in Figure 1. This effect could be partly attributed to the generation of excessive heat and bubbles during protein migration, which could differentially deform the polymeric matrix of the gel and cause irregular migration of the bands of interest. It could also be attributed to possible protein aggregation states or the influence of post-translational modifications such as those already reported for PGHs in some lactic acid bacteria (Rolain et al., 2013), as well as the presence of at least twenty possible phosphorylation sites for this protein and also some potential *N*- and *O*-glycosilation sites (Supplementary Figure S4).

**Figure 2:**
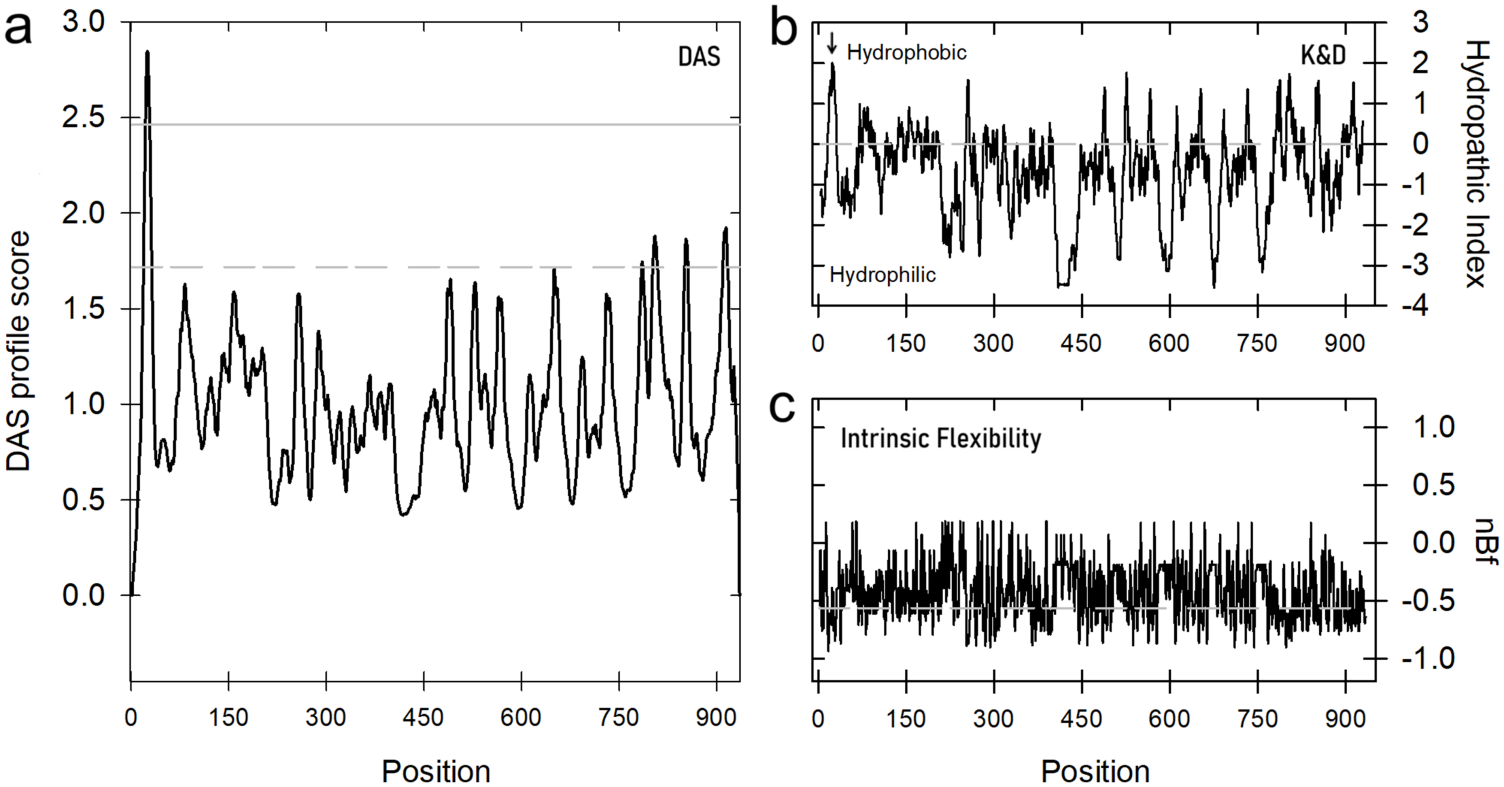
**(a)** Transmembrane (TM) domain prediction using the Dense Alignment Surface (DAS) method. The results of the DAS analysis for the ITV26 PGH suggest the presence of at least one TM domain at the N-terminus of the protein. **(b)** Hydrophobicity plot (Kyte-Doolittle scale) of pediococcal PGH ITV26. The TM domain, rich in Ala residues (*arrow*), has the following sequence: KWAFASIATASLGLVALNTNA. **(c)** Intrinsic structural flexibility profile for the PGH from *P. acidilactici* ITV26 according the FlexiProt software.

To determine whether the PGH enzyme from strain ITV26 has membrane-spanning domains, as suggested by its high activity in the membrane-enriched fraction, the DAS transmembrane prediction server^1^ was used (Cserzö et al., 1997). In the hydrophilicity plots of the 935 amino acid A0AAW8YPL7 sequence (*P. acidilactici* ITV 26), the N-terminal end exhibits a region of high hydrophobicity, suggesting that this protein contains at least one transmembrane domain between residues 15–35 (Figure 2). The analysis of this protein with potent PGH activity was performed using the hydrophobic amino acid scale with ProtScale from ExPASy (Gasteiger et al., 2005; Kyte and Doolittle, 1982). The hydrophobicity score of this protein is consistent with the flexibility profile estimated using the FlexiProt algorithm (García-Morales et al., 2024), with the highly hydrophilic regions being particularly flexible and the hydrophobic regions being more rigid but highlighting the N-terminal end as possibly lipid-anchored to the membrane.

Likewise, as part of the preliminary characterization for this protein, we performed a phylogenetic analysis based on the UPGMA algorithm. Upon submitting several microbial PGHs, the protein present in the *P. acidilactici* ITV26 strain aligns closely with PGHs previously described in *P. pentosaceous* and *P. stilessi*, although more distantly with the PGHs of *Lactilactobacillus sakei* and *Carnobacterium divergens*. In the cladogram shown in Supplementary Figure S5, the sequence of the glycoside hydrolase domain from *S. typhimurium* FlgJ, whose structure is deposited in the PDB (5dn4, 5dn5), was used as the coherent outgroup.

**Figure 3:**
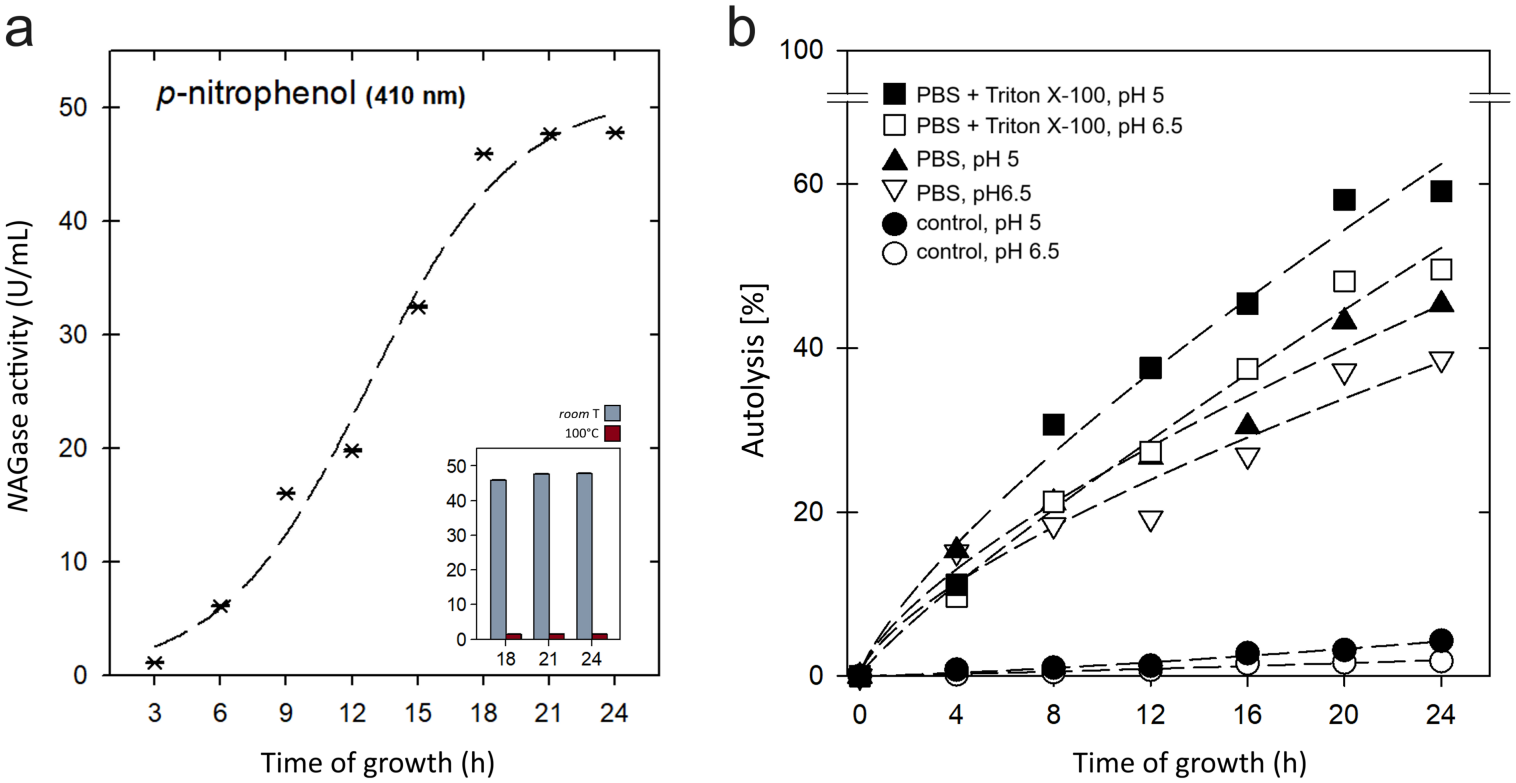
**(a)** Effect of incubation time of *P. acidilactici* ITV26 on the *β*-*N*-acetylglucosaminidase (*N*AGase) activity present in the membrane-rich extract. The optical density at 410 nm of the hydrolyzed product (*p*-nitrophenol) reached a maximum after 18 h of bacterial growth. Data are presented as mean ± standard error of the mean (*n* = 4). Inset shows the loss of function after treatment with 100 °C for 15 min. **(b)** Percentage of autolysis of *P. acidilactici* ITV26. It was assessed over a 24-h period in phosphate buffer (triangles), examining the effects of pH 5.0 (black) and 6.5 (white) and the additional presence of Triton X-100 (squares). A heat-denatured enzyme control (100 °C for 15 min, circles) was also included.

With this information, we decided to evaluate the specific *N*AGase activity present in the enriched extracts from the membrane fraction of *P. acidilactici* ITV26. To do this, we used the hydrolytic release assay of *p*-nitrophenol from the artificial substrate 4-NP-Glc*N*Ac, which simulates the structure of Glc*N*Ac, present in bacterial cell walls. The results shown in Figure 3a indicate that the membrane extract possesses strong *N*AGase activity, which reaches a maximum starting from 18 h of bacterial growth, with an average of 47.15 U/mL up to 24 h. It was also observed that *N*AGase activity is lost after applying a treatment of 100 °C for 15 min, which confirms the enzymatic nature of this activity.

### 3.2 Effect of pH and Triton X-100 on the autolysis of *Pediococcus acidilactici* ITV26

Programmed cell death in bacteria plays an important role in their physiology and cell cycle (Chapot-Chartier and Kulakauskas, 2014). In a next step, the percentage of autolysis of *P. acidilactici* ITV26 was evaluated over time under the effect of two pH conditions (5.0 and 6.5) as well as in the presence of Triton X-100 in phosphate buffer (PBS) (Figure 3b). In the treatments where the ITV26 strain was subjected to 100 °C for 15 min to denature the fraction containing the PGH enzyme, we observed a maximum percentage of autolysis after 24 h of growth of 4.29% at pH 5.0 and 1.80% at pH 6.5. In comparison, 45.29% of the cells were lysed after 24 h in the presence of PBS, pH 5.0, and 38.66% at pH 6.5. Similarly, when this strain grows in the presence of Triton X-100, the highest percentage of autolysis was observed at pH 5.0 with 59.1% at 24 h and, in contrast, only 49.6% at pH 6.5. These experiments clearly show that the autolytic activity of the PGH enzyme of strain ITV26 is strongly dependent on pH, with autolysis being more severe in very acidic conditions. This finding is consistent with what was reported by Ramírez-Nuñez et al. (2011), who demonstrated that, for certain lactic acid bacteria (LAB), stress conditions, including low pH, can increase the rate of autolysis, promoting faster or more complete cell lysis. Our results indicate that the lytic activity of PGH is slightly higher in acidic conditions (pH = 5) and less efficient at pH 6.5, with an alkaline isoelectric point (pI = 9.23). This is consistent with previous reports that have found a correlation between the optimal pH and the isoelectric point in several enzymes, where their activities decrease as the pH approaches the pI value (Götz et al., 1980; Alexov, 2004). This has been interpreted in terms of the basic groups in the enzymatic core structure, which potentially affects the optimal pH by presenting a low acid/base ratio in their aminoacidic composition (Alexov, 2004).

On the other hand, our results are also consistent with those reported by Mora et al. (2003) and Gandhi et al. (2020) for different *Pediococcus* strains, which exhibit diverse degrees of autolysis, ranging between 40 and 90% in PBS buffer at pH 6.5, showing the strain-dependent nature of autolysis in such LAB. In this context, it is also worth mentioning that O-acetylation of PG has a great effect on the autolysis process of *L. plantarum* by inhibiting the *N*-acetylglucosaminidase Acm2, the main autolysin found in this LAB (Bernard et al., 2011).

Regarding the effect of Triton X-100, its role in solubilizing membrane proteins is well documented (Yu et al., 2014). Under our conditions, an acceleration of autolysis was observed at both pH conditions, as it was also noted by Gandhi et al. (2020). This acceleration can be attributed to a synergistic effect between the surfactant action of Triton X-100 and the probable affinity of this nonionic compound to the PG for accelerating the enzymatic activity. This could be explained by referring to the probable peripheral nature of this enzyme in lipid membranes, which has been reported for various endolysins in Gram-positive and Gram-negative bacteria (Nelson et al., 2012) and it is consistent with our bioinformatic prediction (Figure 2). Thus, the membrane solubilization facilitated by Triton X-100 would free these enzymes from their lipid anchors altering the conformation to better expose the active site or facilitating their mobility towards the PG substrate. In that sense, the exposure of new cleavage sites in the exposed PG may be a contributing factor to accelerate autolysis. Consistent with this interpretation, Rolain et al. (2012) and Zaragoza et al. (2009) have demonstrated how membrane permeabilization accelerates bacterial lysis. It is important to highlight that four classes of PGHs have been identified based on their hydrolytic binding specificity to PG (Chapot-Chartier and Kulakauskas, 2014): (1) *N*-acetylmuramidases (muramidases or lysozymes), (2) *N*-acetylglucosaminidases (*N*AGase), (3) N-acetylmuramyl-L-Ala amidases (muramidases), and (4) endopeptidases. Our results suggest that the catalytic glucosaminidase domain we have detected in the sequence of PGH ITV26 as a plausible candidate for this autolytic activity, given that their activity is regulated by the membrane integrity.

Once again, as a control, we tested a heat treatment of 100 °C for 15 min with the aim of denaturing the *N*AGase activity. This method was sufficient to suppress the autolytic capacity of this enzyme (Figure 3b, circles). This result serves as conclusive evidence that the observed autolysis is predominantly an enzymatic process. Scarce autolysis observed under these conditions, particularly at pH 6.5, could be attributed to physical processes such as cell aggregation or the incomplete denaturation of enzymes.

**Figure 4:**
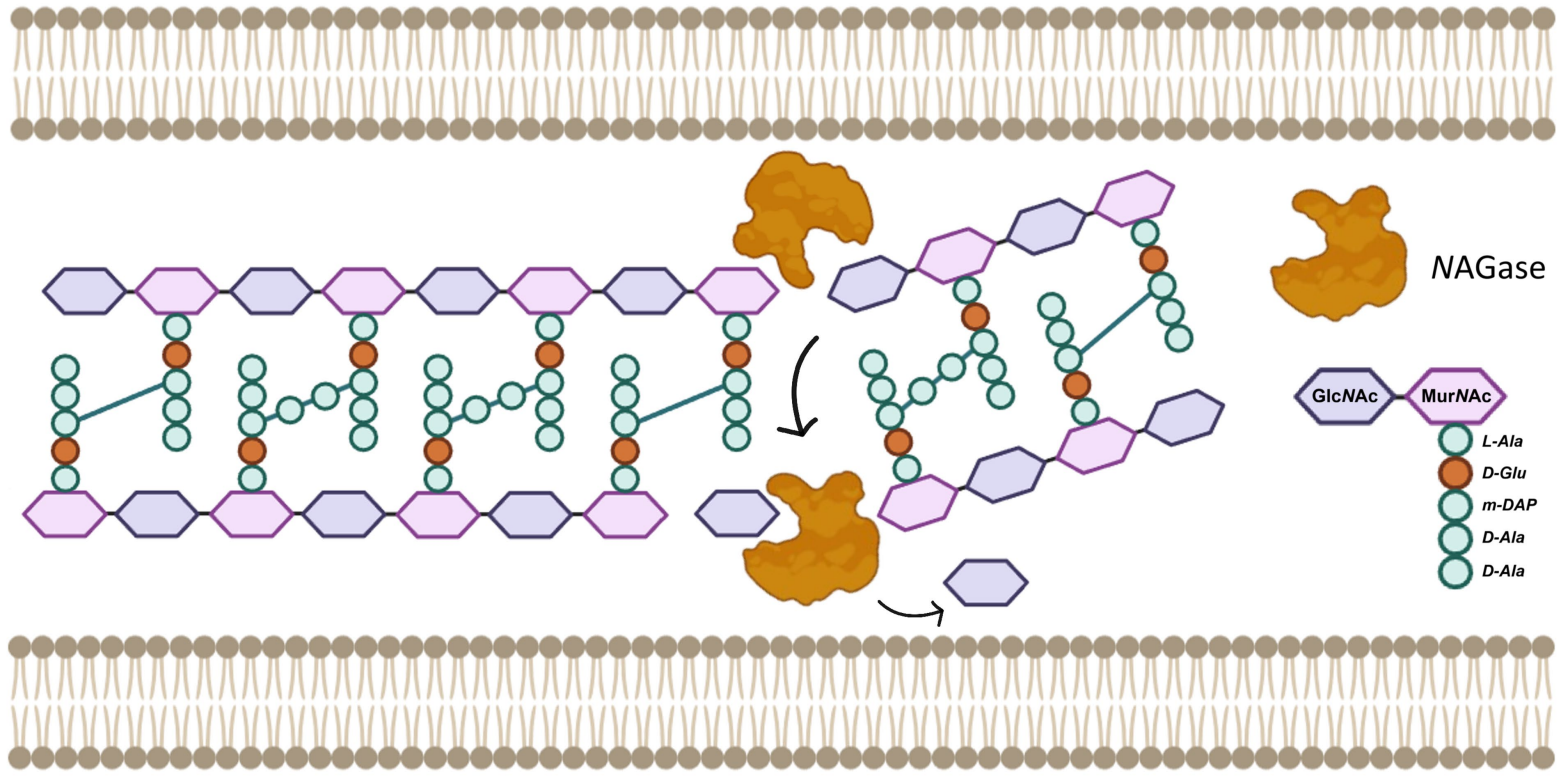
Representative model of the possible mechanism of action of PGH ITV26 as a *N*AGase using Gram-negative bacteria as an example. The *O*-glycosyl hydrolase activity requires a water molecule to cleave the glycosidic bonds, releasing Glc*N*Ac units and cleaving the glycan chains. Although a structural model for this enzyme is beyond the scope of this study, the structure of the non-crosslinked PG monomer shows the disaccharide *N*-acetylglucosamine (Glc*N*Ac) and *N*-acetylmuramic acid (Mur*N*Ac) as well as the specific pentapeptide, typical in Gram-negative bacteria.

This experimental evidence allows us to propose a plausible mechanism that would describe the activity of this *N*AGase against Gram-positive and, to a lesser extent, Gram-negative bacteria as well. For now, we can say that, as has been described for other autolysins (Do et al., 2020; Shaku et al., 2020), PGH ITV26 is a *O*-glycosyl hydrolase (EC 3.2.1.x) with modular architecture, whose *N*AGase catalytic domain is capable of hydrolyzing *β*-(1,4) glycosidic bonds to release Glc*N*Ac and hydro-oligosaccharides (McCarter and Withers, 1994; Davies and Henrissat, 1995). This activity would be sufficient to fragment the murein *sacculus*, compromising its integrity and making the bacteria more susceptible to mechanical and osmotic stress, leading to cell lysis (Figure 4). To the best of our knowledge, in addition to the reports for pediococcal PGHs mentioned above (section 3.1), this study is consistent with other reports where the catalytic activities of other glucosaminidases found in lactobacillales and related bacteria have been characterized (Huard et al., 2004; Eckert et al., 2006), thus strongly consolidating them as enzybiotics with potential pharmacological alternatives.

### 3.3 Antibacterial activity of *Pediococcus acidilactici* ITV26 PGH against gram-positive and gram-negative pathogens

**Figure 5:**
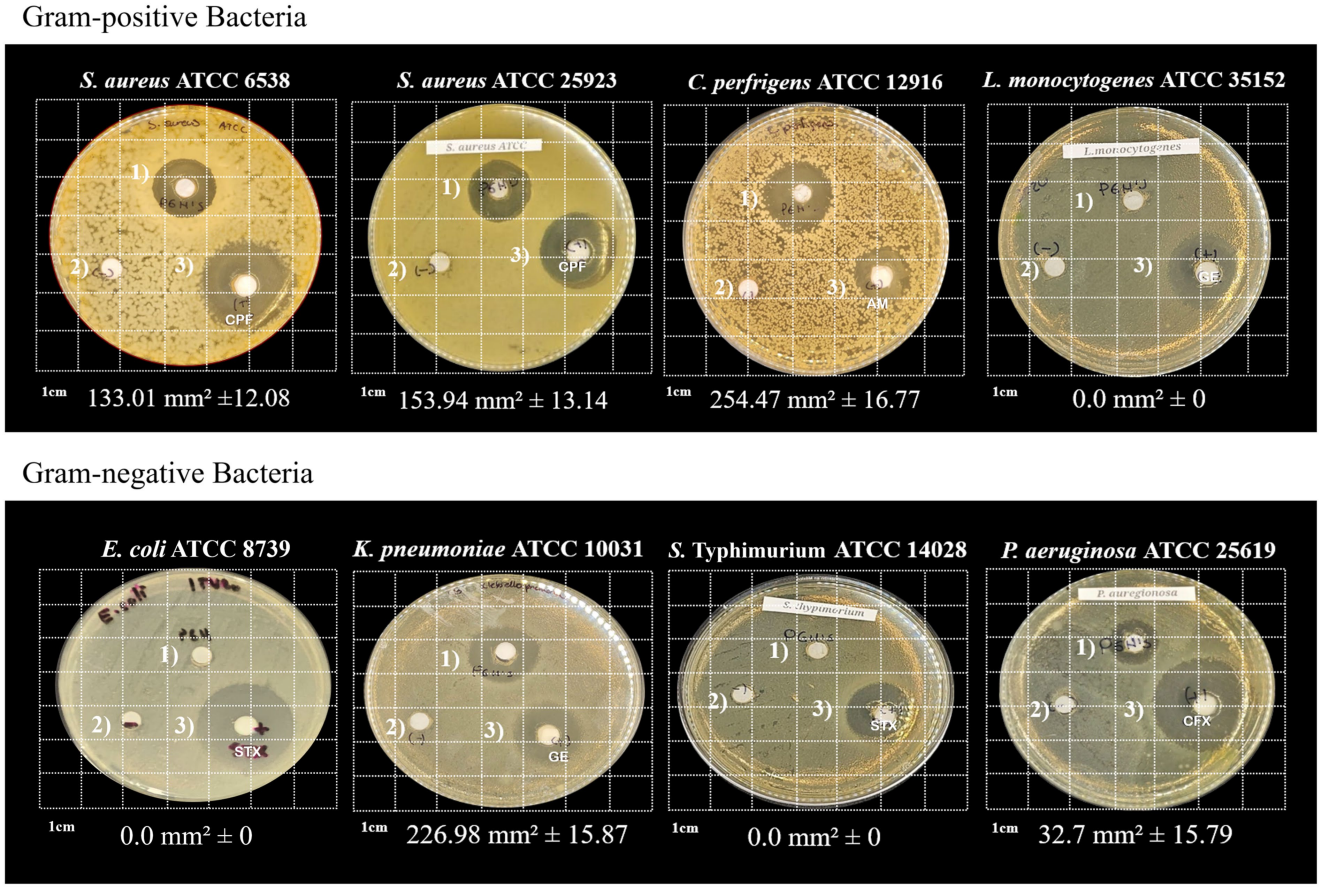
Antimicrobial activity of *P. acidilactici* ITV26 *PGH* against selected Gram-positive and Gram-negative bacteria. 1) PGH enzymatic activity; 2) PGH extraction solution (negative control); and 3) Susceptible antibiotic (positive control): CPF, ciprofloxacin; AM, ampicillin; GE, gentamicin; STX, trimethoprim; CFX, cefotaxime.

The results from the membrane-enriched fraction, where we found the protein close to 110 kDa in the ITV26 strain and which we characterized compositionally and phylogenetically as a PGH, reveal a differential lytic specificity against several pathogenic species, including some from the ESKAPE group (Figure 5). This enzyme demonstrated the ability to lyse Gram-positive cells such as *S. aureus* and *C. perfringens*, as well as some Gram-negative bacteria including *K. pneumoniae*, and *P. aeruginosa*. These results were consistent (*n* = 4) and showed high statistical significance (*p* < 0.001) for the difference between the mean area of enzyme inhibition on the plate dish: 135.36 ± 10.5 mm^2^ (Gram-positive) and 61.31 ± 9.24 mm^2^ (Gram-negative) pathogens. We also detected a certain degree of sensitivity to this PGH in *A. baumannii,* however we are still characterizing the strain to describe these inhibitory effects in detail (*data not shown*). However, the enzyme was inactive against *L. monocytogenes*, *S. typhimurium*, and *E. coli*, highlighting a more severe inhibitory effect on Gram-positive bacteria. According to our sequence analysis, the enzymatic, activity observed in those experiments could correspond to a glucosaminidase, which is capable of hydrolyze the *β*-(1,4)-glycosidic linkages between Glc*N*Ac and *N*-acetylmuramic acid (Mur*N*Ac) that form the glycan backbone of the PG present in the cell envelope (Schleifer and Kandler, 1972).

The finding of lytic activity against *K. pneumoniae* and *P. aeruginosa* is of particular interest. Gram-negative bacteria possess an outer membrane that acts as a formidable permeability barrier, restricting the access of large molecules, including lytic enzymes (Nikaido, 2003). The ability of this PGH from *P. acidilactici* ITV26 to overcome this barrier in some Gram-negative species, but not in others, suggests that outer membrane permeability is a critical factor in antimicrobial resistance and that the high intrinsic flexibility of this protein (Figure 2c) could contribute to optimizing its lytic activity against such phenotypes. The outer membrane in Gram-negative bacteria is composed of glycolipids, primarily lipopolysaccharides (LPS), which play a critical role in the barrier function of this membrane. Since the acyl chains are predominantly saturated, a semisolid, compact packing of these molecules is facilitated. Furthermore, the presence of porins limits the diffusion of hydrophilic molecules larger than 700 Da, making this membrane selective (Nikaido, 2003). This could indicate that a protein with a molecular mass like the one described here (~110 kDa), but with a high hydrophilic profile and highly flexible, could be key to crossing this barrier, which is consistent with the high degree of disorder predicted for this protein (*data not shown*).

In any case, it has been proposed that these PGHs could interact with the bacterial outer membrane and disrupt it through specific interactions by conjugating with diverse chemical moieties capable of permeabilizing it (Nelson et al., 2012). Thus, it remains to be determined if in the membrane-rich extract that we obtain there is some chemical component not yet detected that would facilitate the action of this enzyme. In phage endolysins, it has been shown that their lytic capacity depends on a PG recognition domain, although their access to this molecular mesh in Gram-negative bacteria is not entirely clear (Ghose and Euler, 2020). However, it is hypothesized that an absorption mechanism mediated by another protein domain is key to facilitate the interaction with the outer membrane and access the periplasmic space (Gutiérrez and Briers, 2021). Thus, the high degree of resistance observed in *Salmonella typhimurium* ATCC 14028 and *E. coli* to this PGH highlights the critical role of the outer membrane barrier as a key determinant in the susceptibility of Gram-negative bacteria to external lytic enzymes. Regarding the resistance to the action of this PGH in *L. monocytogenes*, a Gram-positive bacterium, it represents a key point for understanding enzymatic specificity. Despite the absence of an outer membrane, the PG of *L. monocytogenes* is known to exhibit atypical structural modifications, such as teichoic acid glycosylation, deacetylation and O-acetylation of the PG backbone (Meireles et al., 2020; Schulz et al., 2022), which could alter or mask the recognition of the enzyme to its substrate, as reported for the null effect of lysozyme on these bacteria (Schulz et al., 2022).

### 3.4 Scanning electron microscopy

**Figure 6:**
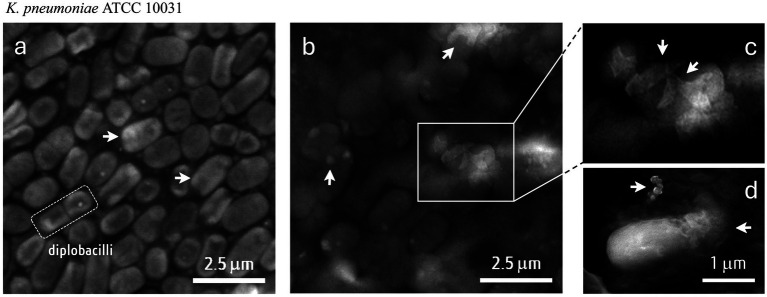
Scanning electron microscopy (SEM) images of *K. pneumoniae* ATCC 10031 untreated **(a)** or treated with PGH ITV26 **(b–d)**. Untreated *K. pneumoniae* (20 K**×**); treated *K. pneumoniae* (9.56 K**×**) **(b,c)** or (45.4 K**×**) **(d)**. The arrows indicate the nature of the polysaccharide capsule: either intact **(a)** or fragmented, forming dense or dispersed aggregates **(b–d)**. PGH concentration was 48 ± 1 μg/mL.

We then repeated the antibacterial assay with a fresh extract rich in the PGH present in the pediococcal membrane fraction, in order to evaluate possible changes in the ultrastructure of each sensitive pathogen by SEM. As shown in Figure 6, changes in the integrity of the capsular polysaccharide, typical of *K. pneumoniae* cells as a clue factor in their virulence (Paczosa and Mecsas, 2016; Xu et al., 2024). Untreated cells show the typical rod shape, frequently found in groups of two cells (dipobacilli), with an intact and smooth surface. In contrast, the morphologies of *K. pneumoniae* cells treated with PGH ITV26 were severely compromised in terms of capsule integrity, with capsular debris observed in the form of high-electron-density aggregates or disaggregated material around the cells (Figure 6d). This indicates that the cells were notably damaged by the PGH. Similar results indicated that the morphologies of *S. aureus* ATCC 6538 and *P. aeruginosa* ATCC 25923 cells treated with this PG hydrolase were modified or destroyed (Supplementary Figure S6). Regarding *C. perfrigens*, the quality of sample fixation was not ideal for obtaining quality SEM images (*data not shown*).

### 3.5 Hemolytic activity of PGH ITV26

The hemolysis assay is often used as a positive control for the characterization of new antibiotics and serves to indicate significant potential toxicity of any drug. *P. acidilactici* ITV26 did not show any hemolytic activity, as it did not cause any detectable zone of inhibition on the cultured red blood cells (Supplementary Figure S7). Therefore, it was classified in the *γ*-hemolysis category, i.e., non-hemolytic, so it can be generally accepted as safe and no pathogenic against human cells (Manglik, 2024).

## 4 Conclusion

In conclusion, we have experimentally demonstrated the antibacterial effects of an enzyme extract associated with the membrane of the bacterium *P. acidilactici*, strain ITV26. The sequence we obtained for the most enriched component in this extract corresponds to a protein of approximately 110 kDa and 935 residues, with a C-terminal region highly homologous to the *N*-acetylglucosaminidase domain present in other bacteria of the *P. acidilactici* group. Our results using a synthetic substrate, analog of *N*-acetylglucosamine, along with the heat inactivation we observed for this enzyme, confirm that we have characterized a PG hydrolase with *N*-acetylglucosaminidase activity. The autolytic activity we detected for this enzyme, which is strongly dependent on the acidity of the medium, could also indicate that the enzyme plays an important role in the cellular physiology of this bacterium. Although this study has not yet elucidated a possible catalytic mechanism for this enzyme, our bioinformatics analysis is consistent and reveals important information for modeling it and proposing a hydrolytic mechanism in a subsequent study. Even so, we have obtained significant electron microscopy evidence that reinforces the idea that this enzyme is capable of altering the integrity of the cell wall in pathogenic bacteria of the ESKAPE group. With all this information, and considering the antimicrobial resistance those bacteria have acquired, the characterization of PG hydrolase enzymes is a highly promising avenue for developing novel therapeutic strategies with enzymes that are harmless to human cells but lethal to several pathogenic bacteria. Validating this enzybiotics potential using *in vivo* animal models to ensure its biosafety in terms of toxicity, pharmacokinetics, and immunogenicity will be a further step towards achieving this aim.

## Data Availability

The data for calculating the intrinsic flexibility of the PGH ITV26 protein, presented in the study, are deposited in the FlexiProt repository, accession number at https://github.com/dballeza/FlexiPROT.

## Footnotes

1
www.sbc.su.se/~miklos/DAS

## Supplementary material

SUPPLEMENTARY FIGURE S1 **(a)** Antibiotic profile of *Pediococcus aidilactici* ITV26 isolated from fresh artisan cheeses and healthy human neonate feces, showing resistance to vancomycin, trimethoprim, ciprofloxacin, and dicloxacillin, but susceptibility to tetracycline, penicillin, erythromycin, clindamycin, cefotaxime, cephalothin, ampicillin, and mildly to gentamicin. **(b)** Bacterial growth of *P. acidilactici* ITV26 in MRS culture medium at 37°C under aerobic conditions.

SUPPLEMENTARY FIGURE S2 **(a)** SDS-PAGE and Zymogram analysis of bacteriocin ITV26. (1) Molecular Weight (MW) marker; (2) Electrophoretic profile of the purified bacteriocin; (3) Zymogram, showing the antimicrobial activity of the purified fraction against *Listeria innocua* AST-062. **(b)** SDS-PAGE of proteins from: (1) *Enterococcus faecium* SP-50; (2) *Lactiplantibacillus plantarum* SP-28; and (3) *P. acidilactici* SP-20; (4) MW marker. **(c)** Results of *S. aureus* ATCC 6538 growth inhibition in the presence of 40.3 ± 4.7 μg/mL of the enzyme extract from the membrane-associated fraction of *P. acidilactici* SP-20 strain. (1) negative control; (2) PGH activity; (3) positive control, CPF (ciprofloxacin).

SUPPLEMENTARY FIGURE S3 Comparison of the amino acid sequences of the PGH from *P. acidilactici* ITV 26 with sequences reported in phylogenetically related microorganisms. These proteins consistently exhibit an *N*-acetylmuramyl-L-Ala amidase domain, an Asn-rich domain, and an *N*-acetylglucosaminidase domain. The enzymes were aligned with homologs from pediococcal lactic acid bacteria using the default settings of Clustal X. Residues marked in color represent site-specific positions for each sequence.

SUPPLEMENTARY FIGURE S4 **(a)** Prediction based on Composition Profile of Patterns (CPP) for N- and O-glycosylation sites in the PGH ITV26 sequence according the GlycoPP server. **(b)** Prediction of phosphorylation sites from ITV26 PGH according the NetPhosBac server. The score of each Ser or Thr residue is plotted in terms of its position in the sequence. If the score is >0.5, the residue constitutes a potential phosphorylation site.

SUPPLEMENTARY FIGURE S5 Similarity cladogram built by the UPGMA method for several PGH-like proteins from lactic acid bacteria. Included as an outgroup is the *S. typhimurium* FlgJ protein, a peptidoglycan hydrolase required for the assembly of bacterial flagella. The evolutionary distance is depicted at the base of each branch.

SUPPLEMENTARY FIGURE S6 SEM images of *S. aureus* ATCC 6538 untreated **(a)** (30.5 K**×**) and treated with PGH ITV26 **(b)**, and untreated *P. aeruginosa* ATCC 25923 **(c)** (34.1 K**×**) and treated **(d)** (30.8 K**×**). The arrows indicate the effects on the typical morphology of each cell after treatment. PGH concentration in each treatment was 48 ± 1 μg/mL. Bar: 1 μm.

SUPPLEMENTARY FIGURE S7 Hemolysis patterns on blood agar. **(a)** Colonies of *P. acidilactici* ITV26 showing γ-hemolysis, characterized by the absence of red blood cell lysis, resulting in growth without any changes to the medium. **(b)** Colonies of *Staphylococcus aureus* ATCC6538, used as a positive control, exhibiting β-hemolysis, with a clear, transparent zone of complete red blood cell lysis around the colonies.
