# Supplementary figures and images for: Antibacterial potential of a new glucosaminidase from Pediococcus acidilactici ITV26 against Gram-negative and Gram-positive pathogens
Source: Front Microbiol. 2025 Dec 29;16:1728482. doi: 10.3389/fmicb.2025.1728482 (PMC12791037; doi:10.3389/fmicb.2025.1728482)

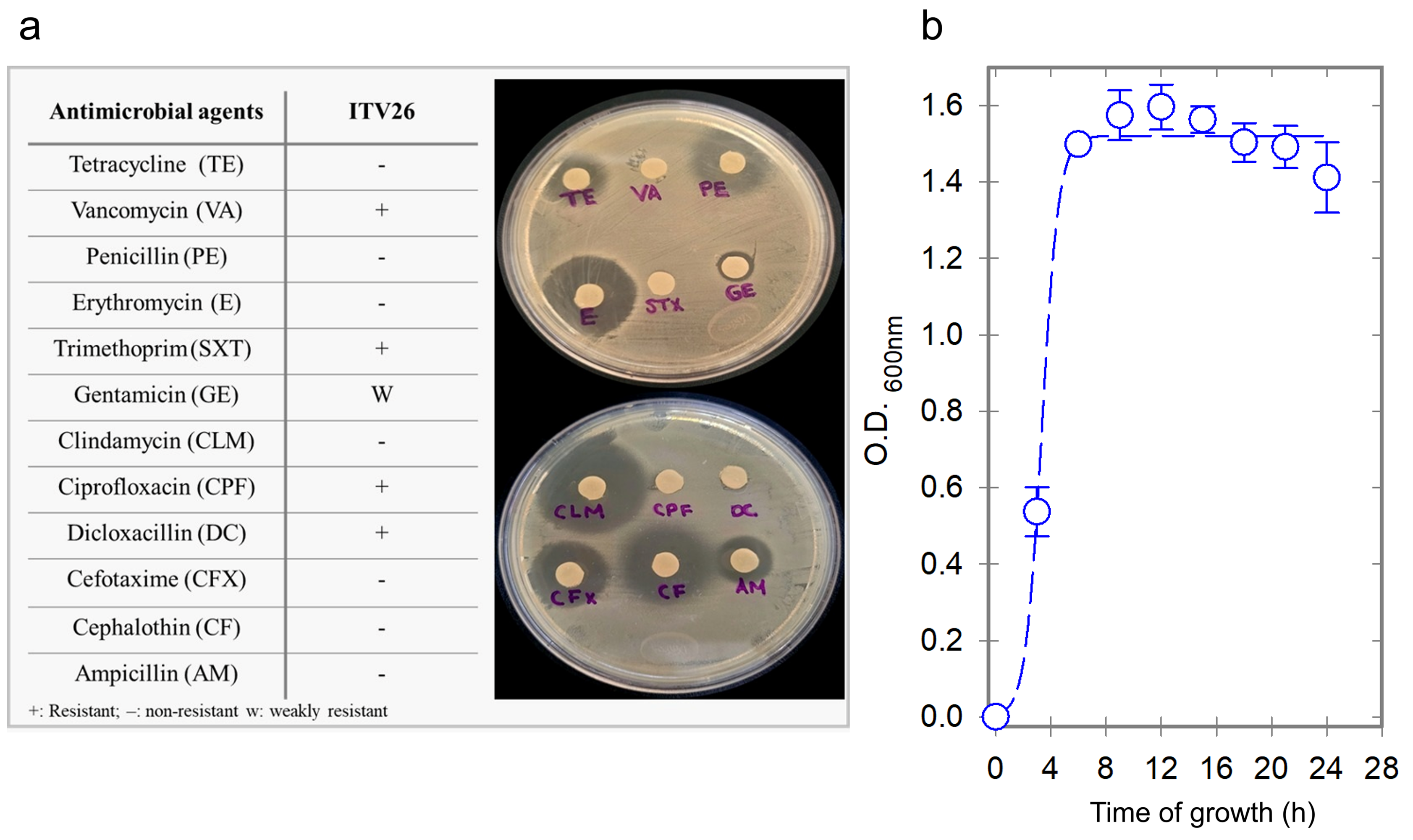

Supplement: SUPPLEMENTARY FIGURE S1 — (a) Antibiotic profile of Pediococcus aidilactici ITV26 isolated from fresh artisan cheeses and healthy human neonate feces, showing resistance to vancomycin, trimethoprim, ciprofloxacin, and dicloxacillin, but susceptibility to tetracycline, penicillin, erythromycin, clindamycin, cefotaxime, cephalothin, ampicillin, and mildly to gentamicin. (b) Bacterial growth of P. acidilactici ITV26 in MRS culture medium at 37°C under aerobic conditions. [file Image_1.TIF]

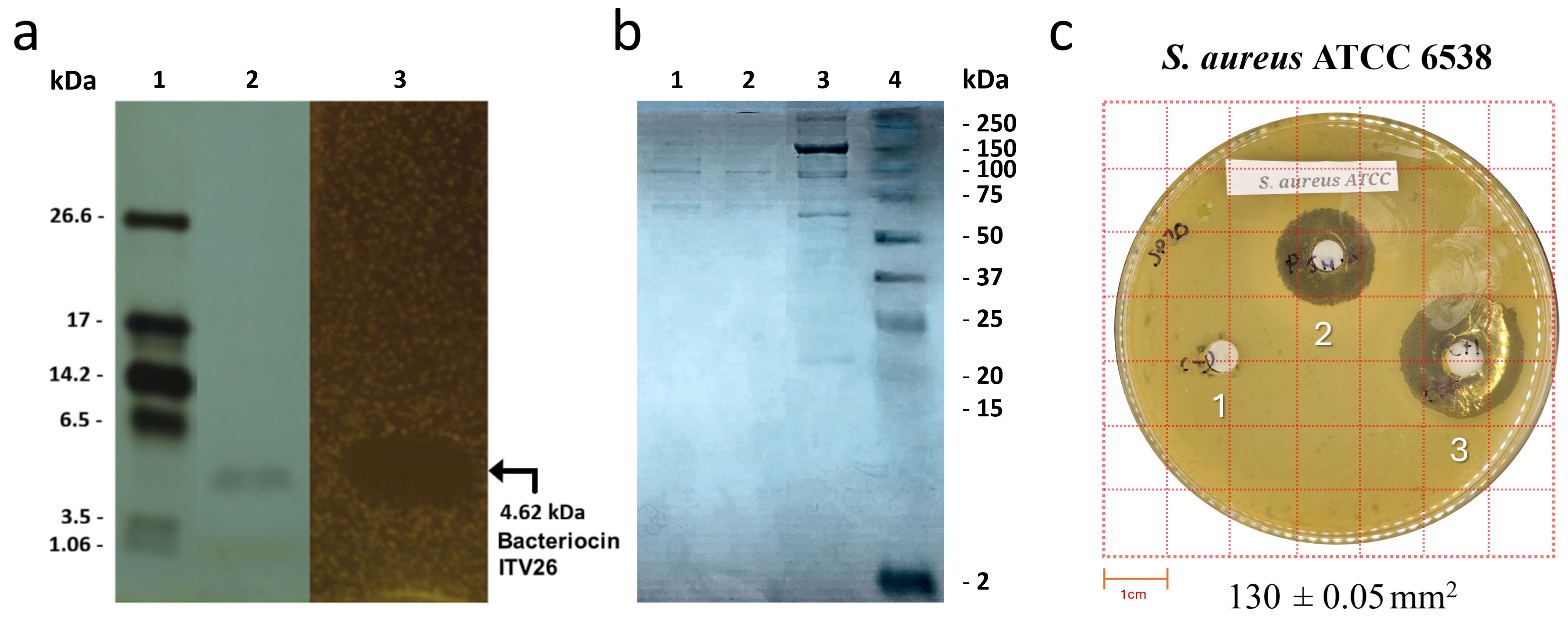

Supplement: SUPPLEMENTARY FIGURE S2 — (a) SDS-PAGE and Zymogram analysis of bacteriocin ITV26. (1) Molecular Weight (MW) marker; (2) Electrophoretic profile of the purified bacteriocin; (3) Zymogram, showing the antimicrobial activity of the purified fraction against Listeria innocua AST-062. (b) SDS-PAGE of proteins from: (1) Enterococcus faecium SP-50; (2) Lactiplantibacillus plantarum SP-28; and (3) P. acidilactici SP-20; (4) MW marker. (c) Results of S. aureus ATCC 6538 growth inhibition in the presence of 40.3 ± 4.7 μg/mL of the enzyme extract from the membrane-associated fraction of P. acidilactici SP-20 strain. (1) negative control; (2) PGH activity; (3) positive control, CPF (ciprofloxacin). [file Image_2.TIF]

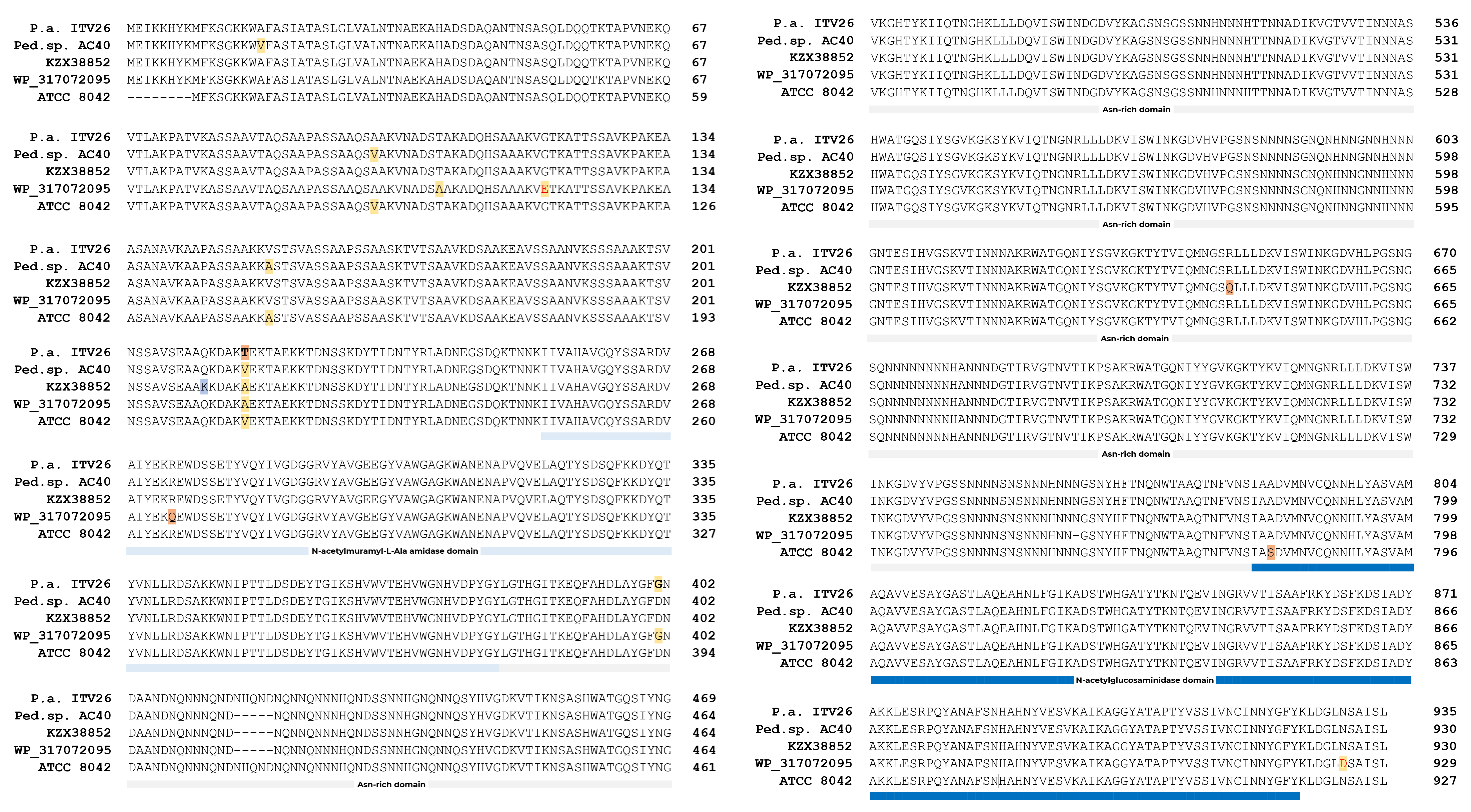

Supplement: SUPPLEMENTARY FIGURE S3 — Comparison of the amino acid sequences of the PGH from P. acidilactici ITV 26 with sequences reported in phylogenetically related microorganisms. These proteins consistently exhibit an N-acetylmuramyl-L-Ala amidase domain, an Asn-rich domain, and an N-acetylglucosaminidase domain. The enzymes were aligned with homologs from pediococcal lactic acid bacteria using the default settings of Clustal X. Residues marked in color represent site-specific positions for each sequence. [file Image_3.TIF]

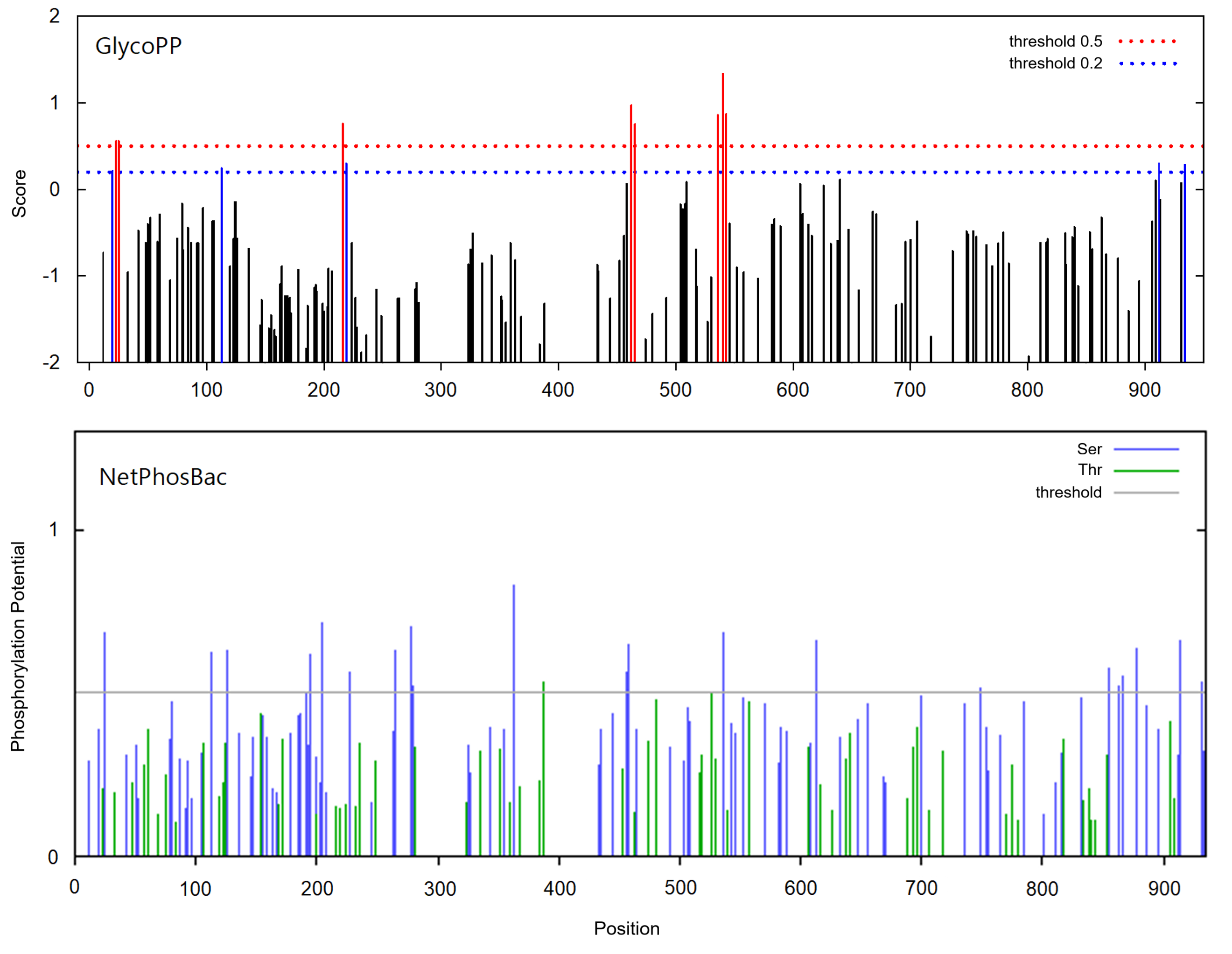

Supplement: SUPPLEMENTARY FIGURE S4 — (a) Prediction based on Composition Profile of Patterns (CPP) for N- and O-glycosylation sites in the PGH ITV26 sequence according the GlycoPP server. (b) Prediction of phosphorylation sites from ITV26 PGH according the NetPhosBac server. The score of each Ser or Thr residue is plotted in terms of its position in the sequence. If the score is >0.5, the residue constitutes a potential phosphorylation site. [file Image_4.TIF]

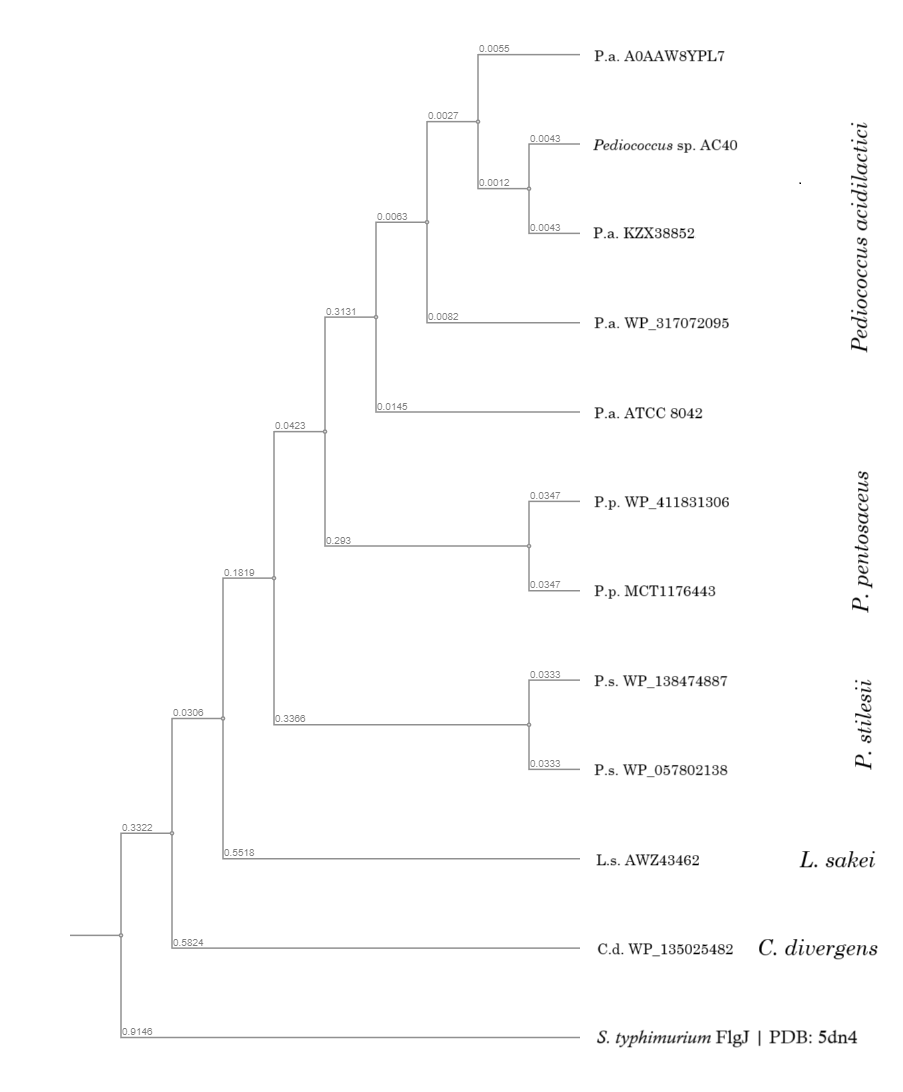

Supplement: SUPPLEMENTARY FIGURE S5 — Similarity cladogram built by the UPGMA method for several PGH-like proteins from lactic acid bacteria. Included as an outgroup is the S. typhimurium FlgJ protein, a peptidoglycan hydrolase required for the assembly of bacterial flagella. The evolutionary distance is depicted at the base of each branch. [file Image_5.TIF]

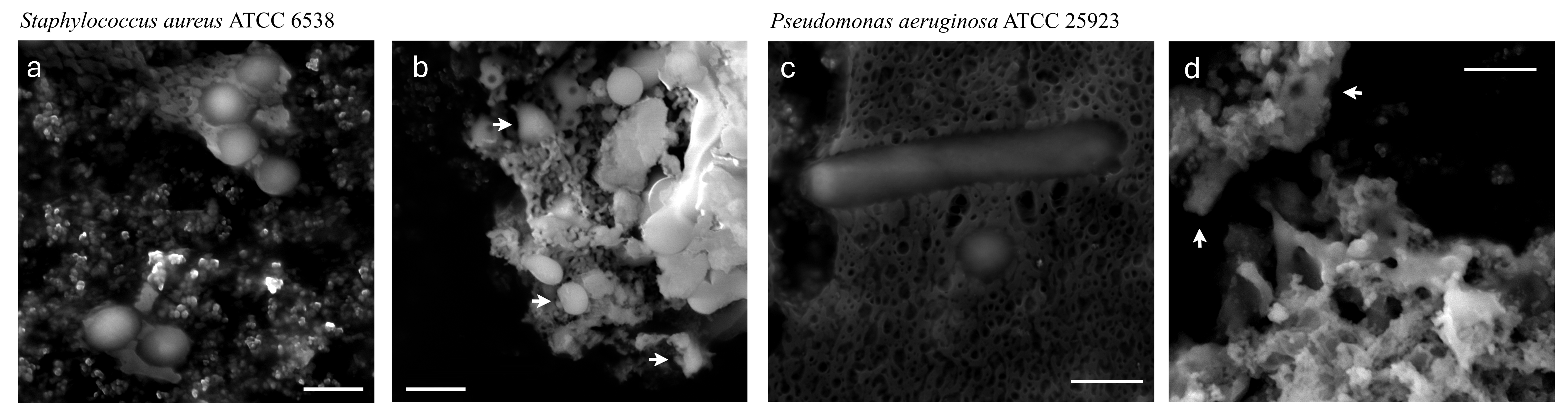

Supplement: SUPPLEMENTARY FIGURE S6 — SEM images of S. aureus ATCC 6538 untreated (a) (30.5 K×) and treated with PGH ITV26 (b), and untreated P. aeruginosa ATCC 25923 (c) (34.1 K×) and treated (d) (30.8 K×). The arrows indicate the effects on the typical morphology of each cell after treatment. PGH concentration in each treatment was 48 ± 1 μg/mL. Bar: 1 μm. [file Image_6.TIF]

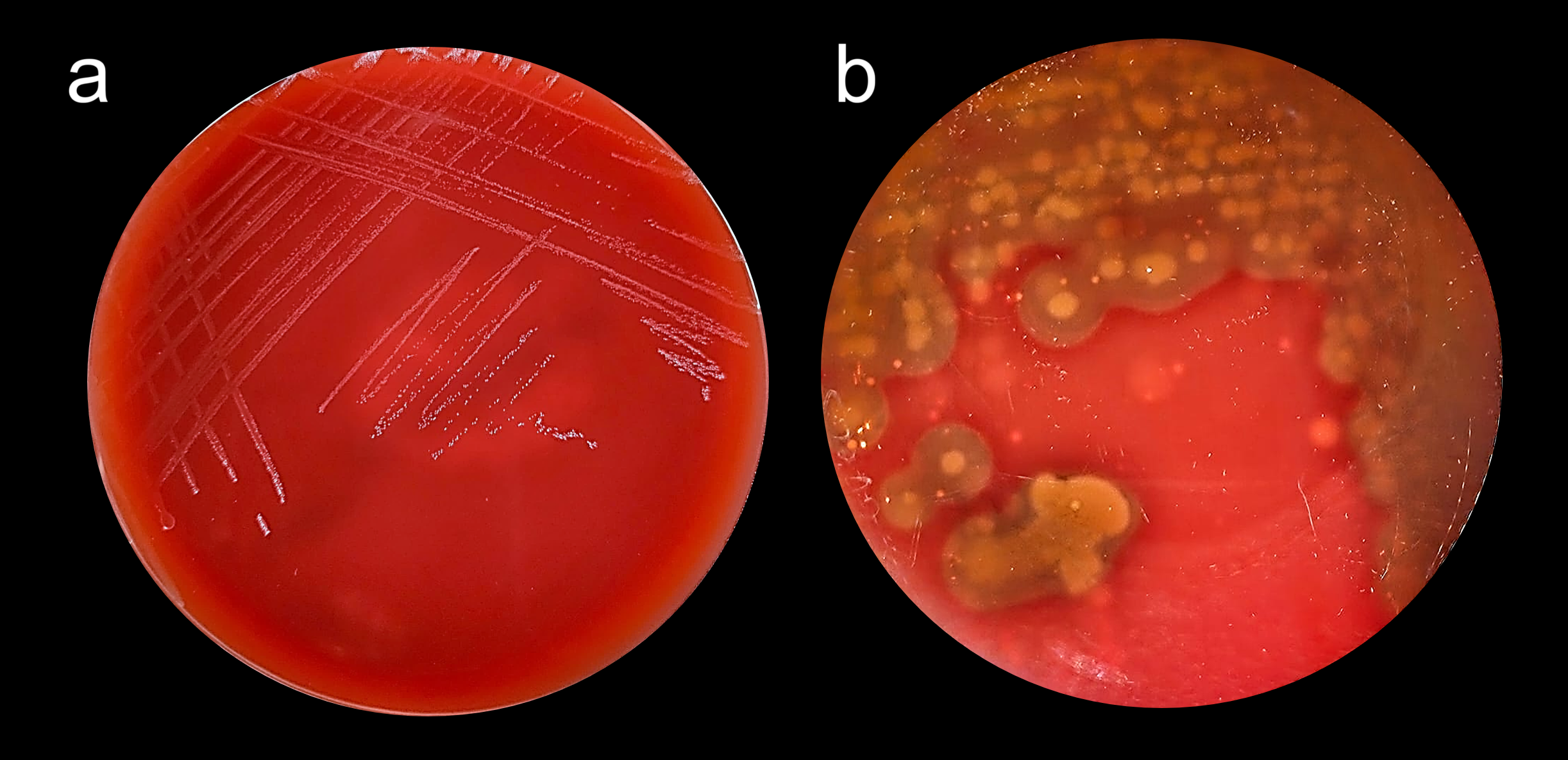

Supplement: SUPPLEMENTARY FIGURE S7 — Hemolysis patterns on blood agar. (a) Colonies of P. acidilactici ITV26 showing γ-hemolysis, characterized by the absence of red blood cell lysis, resulting in growth without any changes to the medium. (b) Colonies of Staphylococcus aureus ATCC6538, used as a positive control, exhibiting β-hemolysis, with a clear, transparent zone of complete red blood cell lysis around the colonies. [file Image_7.TIF]
